# Enhanced Biofilm Formation and Membrane Vesicle Release by *Escherichia coli* Expressing a Commonly Occurring Plasmid Gene, *kil*

**DOI:** 10.3389/fmicb.2018.02605

**Published:** 2018-11-07

**Authors:** Ryoma Nakao, Si Lhyam Myint, Sun Nyunt Wai, Bernt Eric Uhlin

**Affiliations:** ^1^Department of Molecular Biology, The Laboratory for Molecular Infection Medicine Sweden, Umeå Centre for Microbial Research, Umeå University, Umeå, Sweden; ^2^Department of Bacteriology I, National Institute of Infectious Diseases, Tokyo, Japan

**Keywords:** *Escherichia coli*, bacterial biofilms, membrane vesicles, *kil*, ColE1 plasmids

## Abstract

*Escherichia coli* is one of the most prevalent microorganisms forming biofilms on indwelling medical devices, as well as a representative model to study the biology and ecology of biofilms. Here, we report that a small plasmid gene, *kil*, enhances biofilm formation of *E. coli*. The *kil* gene is widely conserved among naturally occurring colicinogenic plasmids such as ColE1 plasmid, and is also present in some plasmid derivatives used as cloning vectors. First, we found that overexpression of the *kil* gene product dramatically increased biofilm mass enriched with extracellular DNA in the outer membrane-compromised strain RN102, a deep rough LPS mutant *E. coli* K-12 derivative. We also found that the *kil*-enhanced biofilm formation was further promoted by addition of physiologically relevant concentrations of Mg^2+^, not only in the case of RN102, but also with the parental strain BW25113, which retains intact core-oligosaccharide LPS. Biofilm formation by *kil*-expressing BW25113 strain (BW25113 *kil^+^*) was significantly inhibited by protease but not DNase I. In addition, a large amount of proteinous materials were released from the BW25113 *kil^+^* cells. These materials contained soluble cytoplasmic and periplasmic proteins, and insoluble membrane vesicles (MVs). The *kil*-induced MVs were composed of not only outer membrane/periplasmic proteins, but also inner membrane/cytoplasmic proteins, indicating that MVs from both of the outer and inner membranes could be released into the extracellular milieu. Subcellular fractionation analysis revealed that the Kil proteins translocated to both the outer and inner membranes in whole cells of BW25113 *kil^+^*. Furthermore, the BW25113 *kil^+^* showed not only reduced viability in the stationary growth phase, but also increased susceptibility to killing by predator bacteria, *Vibrio cholerae* expressing the type VI secretion system, despite no obvious change in morphology and physiology of the bacterial membrane under regular culture conditions. Taken together, our findings suggest that there is risk of increasing biofilm formation and spreading of numerous MVs releasing various cellular components due to *kil* gene expression. From another point of view, our findings could also offer efficient MV production strategies using a conditional *kil* vector in biotechnological applications.

## Introduction

Biofilms are communities of microorganisms that attach to each other and onto biotic and abiotic surfaces. In the clinical setting, medical device-associated infections triggered by biofilm formation are now an emerging problem owing to their resistance to antibiotics, biocides, and host immunity. In addition, antibiotic-resistant bacteria have become a widespread threat to public health on a global scale (World Health Organization[WHO], 2015). Therefore, it is medically important to reveal potential, perhaps cryptic determinants or factors involved in biofilm formation and to elucidate any novel mechanism(s) by which bacteria develop biofilms.

*Escherichia coli* is the most prevalent microorganism that causes catheter-associated urinary tract infections as well as a representative model for studies of bacterial biofilms (Sharma et al., 2016). Several surface-located bacterial appendages of *E. coli*, such as flagella, antigen 43 (Ag43), curli fibers, type I fimbriae, and conjugation pili, are shown to be involved in the biofilm formation (Pratt and Kolter, 1998; Vidal et al., 1998; Danese et al., 2000; Ghigo, 2001; Sherlock et al., 2006). Bacterial autolysis and resultant extracellular release of DNA (eDNA) also serve a crucial role in the initial attachment and biofilm formation by many bacteria (Allesen-Holm et al., 2006; Harmsen et al., 2010; Lappann et al., 2010; Nakao et al., 2012). In addition, the ubiquity of membrane vesicles (MVs), spherical nanoscale proteoliposomes released from biofilm-associated bacteria, has been confirmed by observations of biofilms from a variety of natural and laboratory settings; therefore, MVs are considered common biofilm constituents (Schooling and Beveridge, 2006). MVs contain membrane proteins, lipopolysaccharide, fimbriae, peptidoglycan, nucleic acids, and various periplasmic proteins (Kadurugamuwa and Beveridge, 1995; Wai et al., 2003a; Bonnington and Kuehn, 2014). Consequently, a variety of virulence factors and immunodominant antigens are apparently sorted into MVs. Therefore, MVs not only play a wide array of roles in pathogenesis and immune modulation in many bacteria, but also are offering the applicability of MVs in uses as vaccine antigens (Nakao et al., 2016; Schorey and Harding, 2016) as well as for drug delivery as carriers (Jain and Pillai, 2017; Kim et al., 2017).

The ColE1 plasmid is a naturally occurring colicinogenic plasmid that is mobilizable from one bacterial cell to another in the presence of a plasmid with genes mediating bacterial conjugation such as the F plasmid (Chan et al., 1985). ColE1 and ColE1-like plasmids have been widely found in Enterobacteriaceae (Chan et al., 1985; Riley et al., 1994; Yang et al., 2005; Fricke et al., 2008; Holt et al., 2012; Kunne et al., 2012; Wang et al., 2014; Kurylo et al., 2016). Rijavec et al. (2007) reported that 18 percent of the 215 uropathogenic *E. coli* isolates harbored ColE1 or ColE1-like plasmid. In addition, as ColE1 has been a well-studied and well-defined plasmid since the 1970s, ColE1 was frequently used as a basis for plasmid constructs aimed for molecular cloning or gene expression/complementation studies (Sugino and Morita, 1992; House et al., 2004; Saka et al., 2005; Scott et al., 2017). The biology and functions of colicinogenic plasmid such as ColE1 were comprehensively reviewed by Cascales et al. (2007). The colicinogenic property, which is a characteristic of ColE1, is conferred by a cluster of three genes in ColE1: *cea, imm*, and *kil*. The *cea* gene encodes the colicin E1 protein. The *imm* gene encodes an immunity protein that specifically protects ColE1-carrying cells from colicin E1. The *kil* gene encodes a small lipoprotein Kil, which was involved in the release of colicin E1 protein from its producer cells. However, knowledge about the mechanism action of the *kil* gene is limited up to now.

In the present study, we unexpectedly found that biofilm formation by a deep rough LPS mutant of *E. coli*, RN102, was dramatically enhanced by the introduction of a derivative of ColE1. We identified the *kil* gene originating from ColE1, as being responsible for this enhancement of biofilm formation by *E. coli*. Here, we attempt to understand the mechanistic insight into the *kil* gene-enhanced biofilm formation. Our findings also suggest that *kil*-expressing strain provokes extracellular release of proteinous materials together with aberrant MVs during the process of hyper biofilm formation.

## Materials and Methods

### Bacterial Strains, Plasmids, and Culture Conditions

All the *E. coli* strains and plasmids used in this study are listed in Tables 1, 2, respectively. The *E. coli* strains were grown at 37°C in LB or M9 broth and on agar plates. In most of the experiments, *E. coli* strains were grown for 48 h under static conditions, whereas in the growth studies they were also grown under shaking conditions. Ampicillin, carbenicillin, tetracycline, chloramphenicol, kanamycin, and spectinomycin were supplemented at 100, 50, 10, 25, 50, and 50 μg/mL, respectively, when required. MgSO_4_ was also added in the culture broth at concentrations ranging from 0 to 10 mM. *V. cholerae* non-O1 non-O139 strain V52 used for bacterial killing assay was grown in the LB medium. Rifampicin was supplemented at 100 μg/mL for the culture of the strain V52, when required.

**Table 1:** *E. coli* strains used in this study.

*E. coli* strains*^a^*	Relevant genotypes, phenotypes, or selective marker	Source and/or description
BW25113	wild type, K-12 strain, *lacI*^q^ *rrnB*_T14_ Δ*lacZ*_WJ16_ *hsdR514* Δ*araBAD*_AH33_ Δ*rhaBAD*_LD78_	NIG collection (Japan)
RN101	Δ*waaC*, BW25113 derivative, Hep-deficient LPS core oligosaccharides.	Nakao et al., 2012
RN102	Δ*hldE*, BW25113 derivative, Hep-deficient LPS core oligosaccharides.	Nakao et al., 2012
RN103	Δ*waaF*, BW25113 derivative, LPS core oligosaccharides, which retains only 2 KDO and 1 Hep.	Nakao et al., 2012
RN104	Δ*waaG*, BW25113 derivative, LPS which lacks outer-core oligosaccharides, but retains intact inner-core oligosaccharides.	Nakao et al., 2012
RN105	Δ*waaL*, BW25113 derivative, LPS which retains intact core oligosaccharides.	Nakao et al., 2012
RN107	Δ*galE*, BW25113 derivative, Intact LPS core-oligosaccharides or core lacking galactose.	Nakao et al., 2012
Δ*pldA*	JW3749 (NIG ID Number), BW25113 derivative, outer membrane phospholipase A deficient. Km^r^	NIG collection (Japan)
RN110	*flhD*::Tn*5*, BW25113 derivative, regulator of the flagellar regulon. flagellar deficient. Km^r^	Nakao et al., 2012
NEB turbo	Used for cloning, F’ *proA*^+^*B*^+^ *lacI*^q^ Δ*lacZM15*/*fhuA2* Δ(*lac-proAB*) *glnV gal R*(*zgb-210*::Tn*10*) Tet^s^ *endA1 thi-1* Δ(*hsdS-mcrB*)*5*	New England Biolabs (Ipswich, MA, United States)

*^*a*^Strain BW25113 and the derivatives lack long O-antigen, because the wbbL gene, which encodes a rhamnosyltransferase, is interrupted by the IS5 insertion in the wbbL. Rhamnose is a building block of the O-polysaccharides of LPS.*

**Table 2:** Plasmids used in this study.

Plasmid	Relevant characteristics^a^	Source and/or description
pBR322	Cloning vector, 4.4 kb, Amp^r^ Tc^r^	Bolivar et al., 1977
pACYC184	Cloning vector, 4.2 kb, Cm^r^ Tc^r^	Chang and Cohen, 1978
pMD20-T	T-vector, 2.7 kb, Amp^r^	Takara Bio Inc., (Japan)
pNTR-SD	ColE1 derivative, 8.3 kb, Amp^r^	Saka et al., 2005 NIG collection (Japan)
pNT3(*hldE*)	pNTR-SD derivative containing *hldE* gene under *tac* promoter, utilized for complementation of *hldE* gene, 9.7 kb, Amp^r^	NIG collection (Japan)
pRN021	pBR322 Ω(*mob, esp1*, and *esp2*), containing the 3.8-kb cassette of *mob, eep1*, and *eep2* genes derived from pNTR-SD at *Hin*dIII *and Bam*HI site in pBR322, 7.8 kb, Amp^r^, Tc^s^	This study
pRN022	pACYC184 Ω(*mob, esp1*, and *esp2*), containing the 3.8-kb cassette of *mob* genes, *eep1*, and *eep2* genes derived from pNTR-SD at *Hin*dIII *and Bam*HI sites in pACYC184, 7.6 kb, Cm^r^, Tc^s^	This study
pRN023	pNTR-SD Δ(*mob, eep1*, and *eep2*), 4.8 kb, Amp^r^	This study
pRN024	pRN023 derivative, *kil::Cm*, 5.9 kb, Amp^r^, Cm^r^	This study
pBAD33	Arabinose-inducible expression vector, 5.4 kb, Cm^r^, Tet^r^	Guzman et al., 1995
pRN104	pMD20-T derivative containing sequence of *kil* gene ORF with SD sequence (0.2 kb) from pNTR-SD, 2.9 kb, Amp^r^	This study
pRN109	pBAD33 derivative containing sequence of *kil* gene ORF with SD sequence (0.2 kb) under P_BAD_ promoter, 5.6 kb, Cm^r^, Tet^s^	This study
pMF19	Low copy number expression vector, pEXT21, derivative containing the *wbbL* gene, which can restore the expression of O-antigen in rough LPS, 10.8 kb, Spec^r^	Feldman et al., 1999

*^*a*^Amp^*r*^, ampicillin resistant; Cm^*r*^, chloramphenicol resistant; Spec^*r*^, spectinomycin resistant; Tc^*r*^ or Tc^*s*^, tetracycline resistant or sensitive.*

### Biofilm Formation Assay

Biofilm formation by *E. coli* was assayed using a 96-well flat-bottom polystyrene microtiter plate (Corning 3595, New York, NY, United States) or 5 mL polystyrene tubes (Falcon 352058, BD Labware, Franklin Lake, NJ, United States), as described previously (Nakao et al., 2006), with some modifications. Biofilms were stained with 0.1% crystal violet for 30 min. Crystal violet dye associated with biofilms was eluted with 100% ethanol for 30 min, and was quantified by absorbance at 595 nm. To observe biofilms formed at the interface between air and liquid, bacteria were grown with the coverslips at a stand position in 1 mL broth in a 24-well plate. In advance, the bottom of the wells of a 24-well plate was grooved by a heated loop to keep the coverslips stable at a standing position. To observe biofilms formed at the bottom of the wells, the coverslips were settled at the bottom of the wells during culturing. In every biofilm formation assay, 1 × 10^8^ CFU/mL of *E. coli* was inoculated in broth and grown for 48 h at 37°C. The presence of eDNA in biofilms on coverslips in the wells was examined by staining with ethidium bromide. In confocal laser scanning microscopy (CLSM), biofilms were also stained with a combination of SYTO 9 (Invitrogen, Carlsbad, CA, United States) and BOBO-3 (Invitrogen), as described previously (Seper et al., 2011). In an alternative experiment, the bacterial cells were also stained with a combination of SYTO-9 and propidium iodide (PI, Invitrogen), to discriminate membrane-damaged and -intact cells. The stained samples were examined by using ZEISS LSM 7 live (Carl Zeiss, Oberkochen, Germany) or LSM 700 (Carl Zeiss) equipment. The acquired images were processed by a CLSM Software, ZEN (Carl Zeiss).

### DNA Manipulations

All the DNA manipulations were carried out using standard methods (Sambrook, 2001). The oligonucleotides used in this study are listed in Supplementary Table S1. DNA polymerase (PrimeSTAR HS, Takara Bio Inc., Shiga, Japan) and a T vector (pMD20-T, Takara Bio Inc.) were used for plasmid construction. In each cloning process, an appropriate clone that had the DNA fragment with the correct size and the correct direction was confirmed by PCR and sequencing. In an attempt to identify putative gene(s) in pNTR-SD (Saka et al., 2005) responsible for the effect on biofilm formation, the 3.8-kb *mob-exc1-exc2* DNA region was PCR-amplified using the primer pair *Hin*dIII-Mob-F and *Bam*HI-Mob-R. The *mob-exc1-exc2* DNA cassette was then inserted into pBR322 (Bolivar et al., 1977) and pACYC184 (Chang and Cohen, 1978) at the *Hin*dIII and *Bam*HI sites, resulting in pRN021 and pRN022, respectively. A 4.8 kb fragment of the rest of pNTR-SD was PCR-amplified from pNTR-SD and then the 4.8 kb fragment was self-ligated using a rapid ligation kit (Roche, Penzberg, Germany), resulting in pRN023. The *kil* gene in pRN023 was further disrupted by an insertion of a chloramphenicol resistance cassette at the *Nru*I site located 29 nucleotides from the 5′-end in the *kil* coding sequence, resulting in pRN024. In addition, to construct a *kil*-conditional plasmid, the *kil* gene amplified from pNTR-SD using the primer pair kil2-f and kil2-r (152 bp) was cloned into pMD20-T and then recloned into an arabinose-inducible expression vector pBAD33 (Guzman et al., 1995) at the *Eco*RI and *Hin*dIII sites. The *kil-*conditional clone was named pRN109. A *kil*-FLAG fusion plasmid, named pRN132 was also constructed and the detailed design process of the pRN132 construction was described in Supplementary Materials.

### Subcellular Fractionation of *E. coli*

*Escherichia coli* were cultured for 48 h at 37°C under static conditions. The whole cells and bacterial supernatants were collected by centrifugation at 4,310 × *g* for 20 min at 4°C, and filtered through a 0.45 μm Durapore PVDF (Millipore, Billerica, MA, United States). The supernatant was further subjected to ultra-centrifugation, as previously described (Wai et al., 2003a), with some modifications. Soluble and insoluble fractions of the supernatant were collected as supernatant and pellets after ultra-centrifugation at 100,000 × *g* for 3 h at 4°C in a 45 Ti rotor (Beckman Coulter, Brea, CA, United States). Soluble fraction of the supernatant was concentrated by precipitation using 10% (w/v) trichloroacetic acid (TCA) followed by two washes with 80% acetone to remove TCA. The insoluble fraction (the pellets) of the filtrated supernatant was also collected. The whole cell samples were further subjected to subcellular fractionation by using a differential solubilization technique, as described previously (Wai et al., 2003b). The protein amounts were quantified by the Bradford assay (Bradford, 1976).

### SDS–PAGE and Western Blot

Bacterial cells and the subcellular fractions were subjected to SDS-polyacrylamide gel electrophoresis (SDS–PAGE) followed by an appropriate visualization using a Coomassie brilliant blue (CBB) staining kit (Quick-CBB, Wako Co., Ltd., Osaka, Japan) or a silver staining kit (2D-Silver Stain Reagent II, Cosmo Bio Co., Ltd., Tokyo, Japan), according to the manufacturers’ instructions. Western blot analysis was carried out by standard methods. Rabbit antisera against *E. coli* FliC (Westerlund-Wikstrom et al., 1997) and Ag43 (Beloin et al., 2006) were used as the primary antibodies for Western blot. We also used rabbit antisera against the following subcellular marker proteins: DsbA (localized at periplasm; our collection), Crp (localized at cytoplasm; our collection), OmpC (localized at outer membrane; our collection), and RodZ (localized at inner membrane; purchased from NBRP, NIG, Japan). Horseradish peroxidase (HRP)-labeled anti-rabbit Ig antibody was used as the secondary antibody following these first antibodies. Chemiluminescence was developed by ECL Prime (GE Healthcare Bio-Sciences) or Immobilon ECL Ultra (Millipore, Darmstadt, Germany).

### Transmission Electron Microscopy (TEM)

Transmission electron microscopy analysis was performed as described previously. MV preparations were allowed to adhere to carbon-coated grids for 1 min at room temperature (15–24°C), and then negatively stained with 2% uranyl acetate. The bacterial cells were treated with EEP for 30 min, then prefixed with 2.5% glutaraldehyde and 2% paraformaldehyde in 0.1 M phosphate buffer (pH 7.4) for 2 h at 4°C, and post-fixed in 1% osmium tetroxide. After the preparation by dehydration, the cells were embedded in Epon 812 (TAAB, EM Japan Co., Ltd., Tokyo, Japan). Thin sections were cut and stained with uranyl acetate and lead citrate, and observed with a TEM (HT7700, HITACHI, Hitachi, Japan).

### Flow Cytometry Analysis for the Assessment of Membrane Permeability and Membrane Potential (ΔΨ) of Bacterial Cells

Membrane permeability of the cells of RN102/pNTR-SD was assessed by internalization of two different “cell impermeant” dyes, BOBO-3 (570/602 nm, Thermo Fisher Scientific) and TO-PRO-3 (excitation/emission of at 642/661 nm, Thermo Fisher Scientific). The batch of cells statically grown at 37°C for 3 h was standardized at 1 × 10^6^ CFU/mL in three different FACS buffers as follows: MgSO_4_-free, EDTA-free FACS buffer [10 mM Tris–HCl (pH 8.0), 10 mM glucose], 5 mM MgSO4-supplemented FACS buffer, and 1 mM EDTA-supplemented FACS buffer. These samples were stained with two different “cell-impermeant” dyes; BOBO-3 (1 μM) or TO-PRO-3 (1 μM) and subjected to flow cytometry analysis (FACS Canto II; BD Biosciences, Inc.). Real bacterial particles were discriminated from debris and noise using the forward scatter and side scatter channels (FSC/SSC), which was defined as total bacterial particles, which were collected until they reached 10,000 event. The total bacterial particles were separated on the basis of the difference in fluorescence intensity of TO-PRO-3 or BOBO-3 in a histogram. The bacterial ΔΨ assays were performed as described previously (Yoshimasu et al., 2018) using the whole cells of BW25113/pBAD33 and BW25113/pRN109 (*kil*^+^) collected at different time points of static culture (3, 24, and 48 h). All FACS data were analyzed with FACS Diva software (BD Biosciences).

### Interbacterial Killing in a Type VI Secretion System (T6SS)-Dependent Manner

Bacterial killing assay was performed using *V. cholerae* strain V52 in a T6SS-dependent manner, as described previously (Ishikawa et al., 2012) with some modifications. In brief, *V. cholerae* grown to an OD of 1.8 in the LB medium were mixed at a ratio of 1:3 (vol/vol) with *E. coli* strain BW25113/pBAD33 or BW25113 *kil*^+^ grown to an OD of 1.3 in the LB medium supplemented with 0.02% arabinose. Ten micro liter of this mixture was dropped onto an LB agar plate. After 4 h incubation at 37°C, bacterial growth containing both *V. cholerae* and *E. coli* bacterial cells was harvested from the agar plate. To enumerate colony-forming units (CFUs) of *V. cholerae* and *E. coli*, the serial dilutions of harvested bacterial suspension were inoculated on LB agar containing either rifampicin or chloramphenicol, respectively.

### Phylogenetic Tree Analysis of Kil

The phylogenetic tree was constructed by the neighbor-joining method on the basis of amino acid identities of Kil homologs. Multiple sequence analysis was performed by on-line T-coffee program (version 11.00.8cbe486) served by EMBL-EBI^1^. The accession numbers of the analyzed protein sequences were given in Figure 7.

### Statistical Analysis

Statistical analysis was performed using Prism 7 (GraphPad Software, La Jolla, CA, United States). *P*-values of 0.05 or less were considered to indicate statistical significance.

## Results

### A ColE1 Derivative Plasmid Enhances Biofilm Formation of a Deep Rough LPS Mutant

**FIGURE 1:**
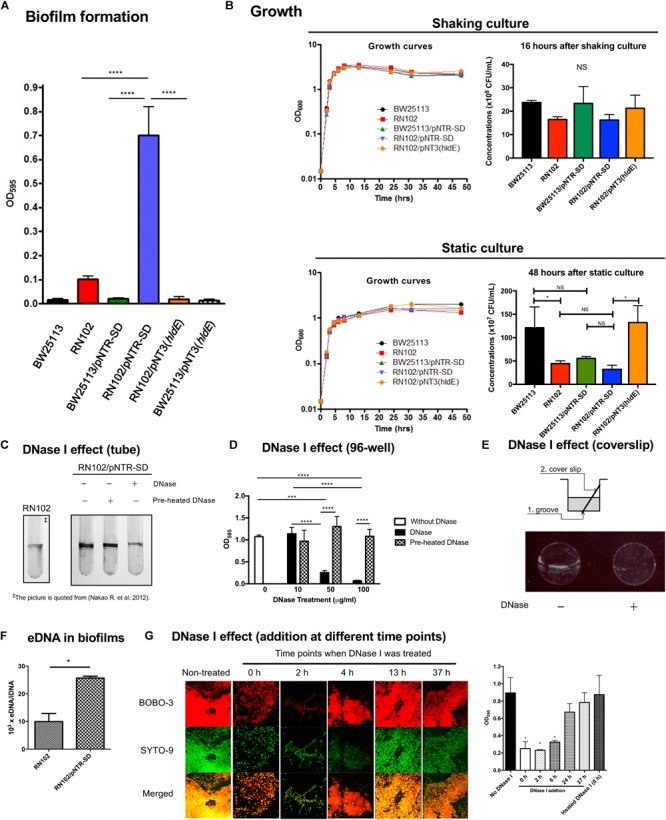
Biofilm formation by *E. coli* strain RN102 harboring pNTR-SD derivatives. **(A)** Biofilm formation by BW25113, RN102, and their plasmid-harboring derivatives. The results are presented as the mean ± SD of four independent experiments. Statistical analysis was performed using one-way ANOVA followed by Dunnett’s test. ^∗∗∗∗^*P* ≤ 0.0001. **(B)** Growth study using five strains as follows: BW25113, RN102, BW25113/pNTR-SD, RN102/pNTR-SD, and RN102/pNT3(*hldE*). Each strain was grown in LB broth under shaking (upper) and static conditions (lower) at 37°C. Absorbance at OD_600_ was measured at different time points during culture for 48 h (left). The CFU of each strain were also determined after 16-h shaking culture and 48-h static culture (right). The results were shown as the mean ± SD of three independent experiments. The CFUs were compared using one-way ANOVA, followed by Dunnett’s test. ^∗^*P* ≤ 0.05. NS, no significant difference among strains. **(C)** Effect of DNase I on biofilm formation by the RN102/pNTR-SD strain in the culture tubes. The RN102/pNTR-SD strain was grown for 48 h under a static condition at 37°C in 5 ml polystyrene tube in the presence or absence of DNase I (100 μg/ml) or preheated DNase I (100 μg/ml). Biofilms were visualized by staining with 0.1% crystal violet. The picture of biofilms of RN102 strain without pNTR-SD plasmid is quoted from Nakao et al. (2012) as a reference control. **(D)** Quantitative assay of biofilms formed on the surfaces of polystyrene. The RN102/pNTR-SD strain was grown in a 96-well polystyrene plate with different concentrations (10, 50, and 100 μg/ml) of DNase I or preheated DNase I or without DNase I for 48 h under static conditions at 37°C. The results are presented as the mean ± SD of three independent experiments. Statistical analysis was performed using one-way ANOVA, followed by Dunnett’s test. ^∗∗∗^*P* ≤ 0.001 and ^∗∗∗∗^*P* ≤ 0.0001. NS, no significant difference among strains. **(E)** The schematic diagrams that describe the setup of the biofilm formation assay to observe the biofilms formed at the interface between air and liquid phases in a well of 24-well plate (top). To keep the coverslip stable in the wells during culture, the bottom of the well was grooved by a heated loop, followed by placing coverslip in the well. After 48 h culture under a static condition at 37°C in a 24-well plate with or without DNase I (100 μg/ml), the biofilms formed at the interface between air and liquid were stained with ethidium bromide (bottom). **(F)** Quantification of eDNA from strains RN102 and RN102/pNTR-SD. The *y*-axis represents the ratio of eDNA to iDNA (eDNA/iDNA). The results are shown as the mean ± SD of three independent experiments. Statistical analysis was performed using Mann-Whitney *U*-test. ^∗^*P* ≤ 0.05. **(G)** DNase I effect on biofilm formation of RN102/pNTR-SD in the course of time. Biofilm images in CLSM using BOBO-3 and SYTO-9 (left) and results of quantitative biofilm assay using 96-well plate biofilm assay (right) after 48-h culture are shown. In the assays, DNase I (100 μg/ml) was added at different time points during the culture period in the assay. The results of the quantitative biofilm assay are shown as the mean ± SD of three independent experiments. Statistical analysis was performed using the Mann-Whitney *U*-test. ^∗^*P* ≤ 0.05 against the biofilm formation level without DNase I treatment (No DNase I).

We have previously reported that one of deep rough LPS mutants of *E. coli*, the *hldE* deletion mutant strain named RN102, increased biofilm formation in comparison with the parental strain BW25113 (Nakao et al., 2012). To confirm that the enhanced biofilm formation was resulting from the disrupted *hldE* gene, a *trans*-complementation test was performed using a *hldE^+^* complementation plasmid clone, pNT3(*hldE*), and the vector control, plasmid pNTR-SD (Saka et al., 2005). RN102 showed increased biofilm formation when compared to BW25113 (Figure 1A), as we previously reported (Nakao et al., 2012). The level of biofilm formation of the strain RN102 was restored by the introduction of pNT3(*hldE*) (Figure 1A), showing that the increased biofilm formation was due to the deletion of *hldE*. Introduction of neither pNT3(*hldE*) nor pNTR-SD altered biofilm formation of BW25113 (Figure 1A). However, surprisingly, RN102 carrying pNTR-SD (RN102/pNTR-SD) enhanced the biofilm formation to a level seven times greater than that of the plasmid-free strain, RN102 (Figure 1A). In growth studies by time-course measurements of OD_600_, BW25113, RN102, and their derivatives (BW25113/pNTR-SD, RN102/pNTR-SD, and RN102/pNT3[*hldE*]) showed similar growth curves in shaking culture, as well as in static culture (Figure 1B). The CFUs of these strains were comparable under shaking culture conditions (Figure 1B). In the static culture, CFUs of RN102 strain are significantly less than those of BW25113 (Figure 1B), in agreement with a previous report about *Salmonella* Typhimurium *hldE* mutant (Jin et al., 2001). Introduction of pNTR-SD into RN102 did not change the CFUs, whereas in *trans*-complementation of *hldE* gene resulted in increase in CFUs (Figure 1B).

### Biofilm Formation by RN102/pNTR-SD Is Dependent on Extracellular DNA (eDNA)

Although the pNTR-SD introduction did not cause obvious growth inhibition in both the BW25113 and RN102 strains, we hypothesized that the hyper-biofilm formation by the RN102/pNTR-SD strain might be due to a combinational effect of this cryptic plasmid and the *hldE* gene mutation, which is known to cause pleiotropic effects (Nakao et al., 2012). To examine the mechanism behind the hyper-biofilm formation, we first examined the biofilm properties in the context of DNase-dependent mechanism. In a clear tube, RN102/pNTR-SD formed matured biofilms mainly at the interface between air and liquid, which was much stronger than those formed by RN102 lacking pNTR-SD (Figure 1C). Even though DNase I was added to the 48-hour-cultured biofilms of RN102/pNTR-SD, there was no effect on the biofilm formation (data not shown). However, the addition of DNase I at the onset of the culturing significantly inhibited biofilm formation of RN102/pNTR-SD, whereas preheated DNase I did not (Figure 1C). Similar results were obtained in the biofilm formation assay on 96-well plates, and a dose-dependent effect of DNase I on biofilm formation was confirmed (Figure 1D). Ethidium bromide staining showed that eDNA was present in the biofilms at the interface between air and liquid phases (Figure 1E). We have also found that pNTR-SD significantly increased the amount of eDNA in the biofilms (Figure 1F). To know the timing when eDNA is required for the biofilm development of RN102/pNTR-SD, the static culture was treated with DNase I at different time points during culturing for 48 h (0, 2, 4, 13, and 37 h). The appearances of 48-hour-old biofilms treated without and with DNase I at each time point were shown in Figure 1G. In the confocal laser scanning microscopic analysis, BOBO-3 was used as an eDNA indicator dye, together with a cell-permeant dye SYTO 9 to counter stain for intracellular nucleic acids (Figure 1G). Owing to the “leaky” phenotype of RN102/pNTR-SD strain (Supplementary Figure S1), BOBO-3 stained not only eDNA, but also the bacterial cells in biofilms (Figure 1G). Nevertheless, the results showed that RN102/pNTR-SD forms matured biofilms when DNase I was untreated or treated at mid or late stage of culture (13 or 37 h, Figure 1G). On the other hand, only small amounts of cells were attached on the surface when DNase I was added at 0 or 2 h, and immature biofilms were observed when DNase I was added at 4 h (Figure 1G). Similar results were obtained in the quantitative biofilm formation assay using 96-well microtiter plates (Figure 1G). Taken together, these findings suggest a substantial contribution of eDNA at an initial attachment stage of the biofilm formation.

**FIGURE 2:**
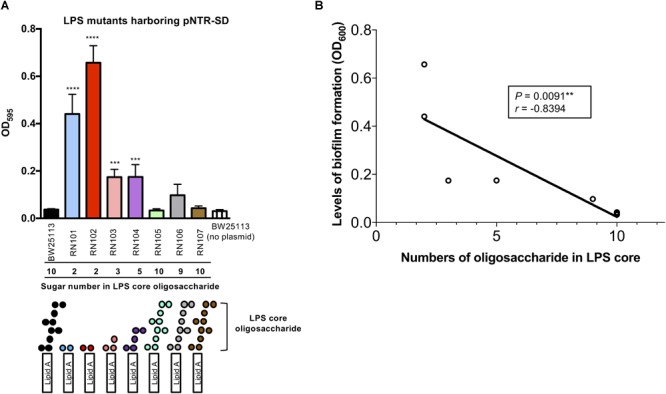
Effect of pNTR-SD on biofilm formation by isogenic *E. coli* strains with different LPS sugar compositions. **(A)** Biofilm formation of isogenic LPS mutants harboring pNTR-SD. Isogenic LPS mutant strains harboring pNTR-SD and the parental BW25113 strain without pNTR-SD were statically grown for 48 h in a 96-well polystyrene plate. Biofilms were visualized by staining with 0.1% crystal violet. The number of LPS core oligosaccharide chains of each strain with LPS structure is shown at the bottom of **(A)**. The results are presented as the mean ± SD of three independent experiments. The levels of biofilm formation by the nine strains were compared using one-way ANOVA followed by Dunnett’s test. ^∗∗∗^*P* ≤ 0.001 against BW25113/pNTR-SD. ^∗∗∗∗^*P* ≤ 0.0001 against BW25113/pNTR-SD. **(B)** The correlation between number of oligosaccharides in LPS core and biofilm formation level is shown. Pearson correlation coefficients revealed the negative correlation with a statistical significance (*P* = 0.0091^∗∗^, *r* = –0.8394).

Next, we examined how pNTR-SD would affect biofilm formation by a series of isogenic LPS mutant strains with different core oligosaccharides compositions (Table 1). The wild type strain (BW25113), RN105, RN106, and RN107 retain 9 or 10 sugars in the core oligosaccharide portions of LPS, whereas the other strains (RN101, RN102, RN103, and RN104) have only 2∼5 sugar numbers in the core-oligosaccharides (Figure 2A). Introduction of pNTR-SD significantly enhanced the biofilm formation in case of four out of eight tested strains with biosynthesis disorders of outer core oligosaccharides of LPS (RN101, RN102, RN103, and RN104) as compared with BW25113 without carrying plasmid (Figure 2A). Pearson’s correlation analysis revealed that the number of LPS core oligosaccharide chain units was inversely correlated to the level of biofilm formation by *E. coli* strains carrying pNTR-SD (Figure 2B), i.e., the highest level of biofilm formation was occurring in the deep rough mutants such as RN101 and RN102.

### The *kil* Gene Is Responsible for the Hyper-Biofilm Formation of RN102/pNTR-SD

**FIGURE 3:**
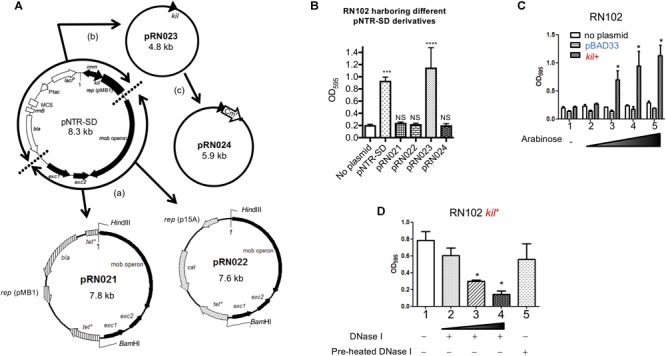
Identification of a cryptic locus in plasmid pNTR-SD responsible for enhanced biofilm formation in the RN102/pNTR-SD strain. **(A)** The scheme of modification of pNTR-SD is shown. The white and black bars (or arrows) in pNTR-SD are indicating regions derived from pJF118HE (Furste et al., 1986) and the ColE1 plasmid (Chan et al., 1985), respectively. First, we conceptually divided pNTR-SD into two parts: the lower right and the upper left divided by the dotted lines shown in the map of pNTR-SD. The lower right fragment was cloned into pBR322 and pACYC184, resulting in pRN021 and pRN022. The upper left fragment was self-ligated, resulting in pRN023. A chloramphenicol-resistance cassette was inserted into the *kil* gene in pRN023, resulting in pRN024. **(B)** Each plasmid shown in **(A)** was introduced into the strain RN102 and their effect on biofilm formation of RN102 was examined. These strains were statically grown for 48 h in a 96-well polystyrene plate. Biofilms were visualized by staining with 0.1% crystal violet. These results are presented as the mean ± SD of three independent experiments. Statistical analysis was performed using one-way ANOVA against a control strain (No plasmid). ^∗∗∗^*P* ≤ 0.001 and ^∗∗∗∗^*P* ≤ 0.0001. NS, no significant difference. **(C)** Biofilm formation of RN102, RN102/pBAD33, and RN102/pRN109 (*kil*^+^) in a 96-well polystyrene plate in the absence or presence of arabinose at different concentrations ranging from 0.0002 to 0.2%. These strains were statically grown for 48 h in a 96-well polystyrene plate. Biofilms were visualized by staining with 0.1% crystal violet. Lane 1, no addition of arabinose; lane 2, 0.0002%; lane 3, 0.002%; lane 4, 0.02%; lane 5, 0.2%. The mean ± SD of results from three independent experiments are shown. Statistical analysis was performed using the Mann-Whitney *U*-test. ^∗^*P* ≤ 0.05, when comparing biofilm formation levels between RN102/pBAD33 and RN102 *kil*^+^ at the same concentration of arabinose. **(D)** RN102/pRN109 (*kil*^+^) was grown for 48 h under static conditions at 37°C in a 96-well polystyrene plate in the absence or present of DNase I at different concentrations. Biofilms were visualized by staining with 0.1% crystal violet. Lane 1, no addition of DNase I; lane 2, addition of DNase I (10 μg/ml); lane 3, addition of DNase I (50 μg/ml); lane 4, addition of DNase I (100 μg/ml); lane 5, addition of pre-heated DNase I (100 μg/ml). The mean ± SD of results from three independent experiments are shown. Statistical analysis was performed using the Mann-Whitney *U*-test. ^∗^*P* ≤ 0.05 against the level of the strain without DNase I treatment.

We next tried to identify the postulated cryptic locus in the pNTR-SD plasmid responsible for biofilm formation. Maps of the pNTR-SD plasmid and the derivatives are shown in Figure 3A. First, we focused on a 3.8 kb region including the *mob* operon and its downstream genes, *exc1* and *exc2*, which encode the elements indispensable for the mobility of pNTR-SD. The 3.8 kb fragment was introduced into two different plasmid vectors, pBR322 (pMB1 *ori*) and pACYC184 (p15A *ori*), resulting in pRN021 and pRN022, respectively. However, the introduction of neither pRN021 nor pRN022 caused any enhancement of biofilm formation by the RN102 (Figure 3B). Next, the pNTR-SD plasmid lacking these mobility elements was constructed by self-ligating the remaining part of pNTR-SD, resulting in pRN023. RN102 carrying pRN023 (RN102/pRN023) displayed a strongly enhanced biofilm formation (Figure 3B). The significant difference between the biofilms formed by RN102/pRN023 and RN102 (lacking any plasmid) was even higher than the difference between RN102/pNTR-SD strain and RN102 (Figure 2B), presumably due to an increase in copy number of the pRN023 plasmid as a result of the reduction of plasmid size from 8.3 kb (pNTR-SD) to 4.8 kb (pRN023), as is the case reported in Smith and Bidochka (1998) and Sambrook (2001). The postulated determinant was revealed after the introduction of insertion mutation in the *kil* gene (pRN024) as it completely abolished the hyper-biofilm phenotype observed in the case of RN102/pNTR-SD or RN102/pRN023 strain (Figure 3B). Furthermore, analysis of the *kil* gene locus, separately cloned into a vector (pBAD33) which allowed for conditional expression induced by arabinose (Guzman et al., 1995), revealed that biofilm formation by the RN102 strain harboring the *kil* expression plasmid, named pRN109 (*kil*^+^), was enhanced in an arabinose-dose-dependent manner, whereas the strain RN102 without plasmid or carrying the vector control plasmid, pBAD33, did not respond to the addition of arabinose at all (Figure 3C). These results clearly demonstrated that the *kil* gene is responsible for the hyper-biofilm formation by the RN102 strain. In addition, we showed the susceptibility of biofilms by the RN102/pRN109 (*kil*^+^) strain to DNase I treatment (Figure 3D), in consistent with the case of the RN102/pNTR-SD strain (Figure 1D). We also introduced the pRN109 (*kil*^+^) plasmid into the BW25113 strain and into another outer membrane-compromised Δ*pldA* strain (Supplementary Figure S2). The biofilm formation by the Δ*pldA* strain, but not by the BW25113 strain, was enhanced after the introduction of the *kil*-expression clone (Supplementary Figure S2), confirming the relationship between defect in outer membrane integrity and susceptibility to the effect of *kil* gene.

### BW25113 Also Enhanced *kil*-Dependent Biofilm Formation in the Presence of a Physiologically Relevant Concentration of Mg^2+^

**FIGURE 4:**
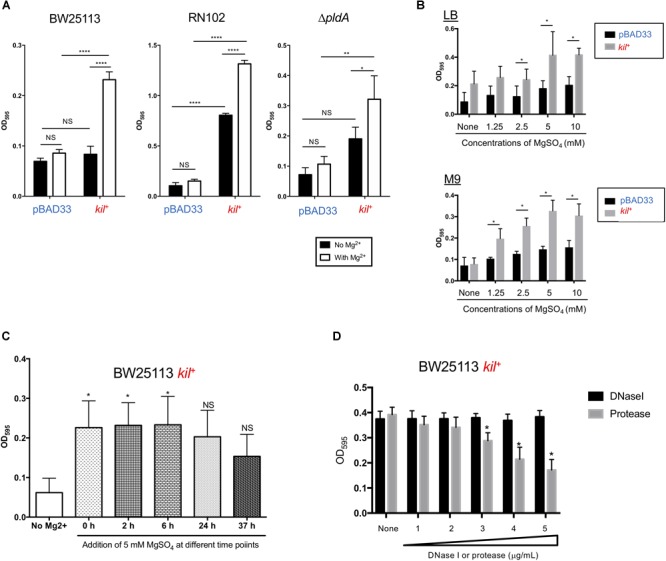
The *kil* gene affects biofilm formation of BW25113 in the presence of Mg^2+^. **(A)** BW25113, RN102, and the Δ*pldA* derivative harboring either pBAD33 or pRN109 (*kil*^+^) were grown in LB containing 0.02% arabinose, with or without 5 mM Mg^2+^. The biofilm formation was quantified using the 96-well biofilm formation assay. The results are expressed as the mean ± SD of three independent assays. Statistical analysis was performed using one-way ANOVA, followed by Dunnett’s test. ^∗∗∗∗^*P* ≤ 0.0001, ^∗∗^*P* ≤ 0.01, and ^∗^*P* ≤ 0.05. NS, no significant difference. **(B)** Biofilm formation by BW25113 harboring pBAD33 and pRN102 (*kil*^+^) was investigated after growth in LB (top graph) or M9 broth (lower graph) supplemented with 0.02% arabinose and MgSO_4_ at different concentrations. The results are expressed as the mean ± SD of three independent assays. Statistical analysis was performed using the Mann-Whitney *U*-test. ^∗^*P* ≤ 0.05, when compared the levels of BW25113 harboring pBAD33 and pRN102 (*kil*^+^) at each MgSO_4_ concentration. **(C)** MgSO_4_ effect on biofilm formation of BW25113 *kil*^+^ in the course of time. Shown are results of quantitative biofilm assay using 96-well plate biofilm assay after 48-h culture. In the assays, MgSO_4_ was added to media at the final concentration of 5 mM at different time points indicated during the culture period in the assay. Results are shown as the mean ± SD of three independent experiments. Statistical analysis was performed using the Mann-Whitney *U*-test. ^∗^*P* ≤ 0.05 against the biofilm formation level without MgSO_4_ addition (No Mg^2+^). **(D)** Biofilm formation by BW25113 *kil*^+^ in the presence of DNase I or protease. BW25113/pRN109 was grown for 48 h under static conditions at 37°C in LB containing 0.02% arabinose in a 96-well polystyrene plate with different concentrations of DNase I or protease. DNase I and protease were used at the following concentrations: lane 1, 31.25 μg/ml; lane 2, 62.5 μg/ml; lane 3, 125 μg/ml; lane 4, 250 μg/ml; lane 5, 500 μg/ml. Biofilms were visualized by staining with 0.1% crystal violet. The results are expressed as the mean ± SD of three independent assays. Statistical analysis was performed using Mann-Whitney *U*-test. ^∗^*P* ≤ 0.05, when compared with the case without treatment (none).

Several reports have suggested a relationship of biofilm formation to a physiologically relevant concentration of Mg^2+^ (∼1 mM in human blood and ∼5 mM in human urine) (Banin et al., 2006; Robertson et al., 2012; He et al., 2016). We therefore tested the effect of 5 mM Mg^2+^ on biofilm formation by the three strains BW25113, RN102, and Δ*pldA* carrying pBAD33 or pRN109 (*kil*^+^) (Figure 4A). As expected, all these strains more or less showed increased levels of biofilm formation in the presence of 5 mM MgSO_4_ (Figure 4A). Of note, in the presence of 5 mM Mg^2+^, BW25113 gained a 2.5-fold further increase in the biofilm formation after the introduction of *kil* gene (Figure 4A, BW25113). A dose-dependent effect of Mg^2+^ on the biofilm formation by the *kil*-expressing strain (BW25113 *kil*^+^) strain was observed in the cultures using a minimum defined medium M9, as well as the LB medium (Figure 4B). Similar results were obtained when MgCl_2_ was used in place of MgSO_4_ (data not shown). We also found that biofilms formed by the BW25113 *kil*^+^ strain were mainly present at the bottom of wells or tubes (data not shown), unlike the case of the RN102 strain harboring the *kil* plasmid, in which its biofilms were formed at the interphase between air and liquid (Figure 1C). Addition of Mg^2+^ altered neither the expression level of flagella protein FliC (Supplementary Figure S3), nor the motility (data not shown) of the BW25113/pBAD33 and BW25113 *kil*^+^ strains, suggesting that flagella expression/motility was unaffected during the Mg^2+^-dependent biofilm formation. The expression level of Ag43 rather decreased in a Mg^2+^ dose-dependent manner, indicating that Ag43 expression was not needed for the enhanced biofilm formation in the presence of Mg^2+^ (Supplementary Figure S3). We have also monitored the utilization of Mg^2+^ in the course of time (Figure 4C). Mg^2+^ significantly enhanced biofilm formation when Mg^2+^ was added to culture media at log phase, but not stationary phase (Figure 4C). Furthermore, we tested the susceptibility of biofilm formation of BW25113 *kil*^+^ strain to DNase I and protease treatments (Figure 4D). The enhanced biofilm formation was partially inhibited by protease in a dose-dependent manner, but not by DNase I at all (Figure 4D), unlike the case of biofilm formation of RN102 *kil*^+^ strain (Figure 3D). Similar results were obtained in the case of a flagella-deficient mutant strain harboring the *kil* expression plasmid (*flhD^-^ kil*^+^) (Supplementary Figure S4). Thus, we concluded that flagella expression was not involved in the protease-dependent biofilm formation inhibition.

### BW25113 *kil*^+^ Produces High Amounts of MVs

**FIGURE 5:**
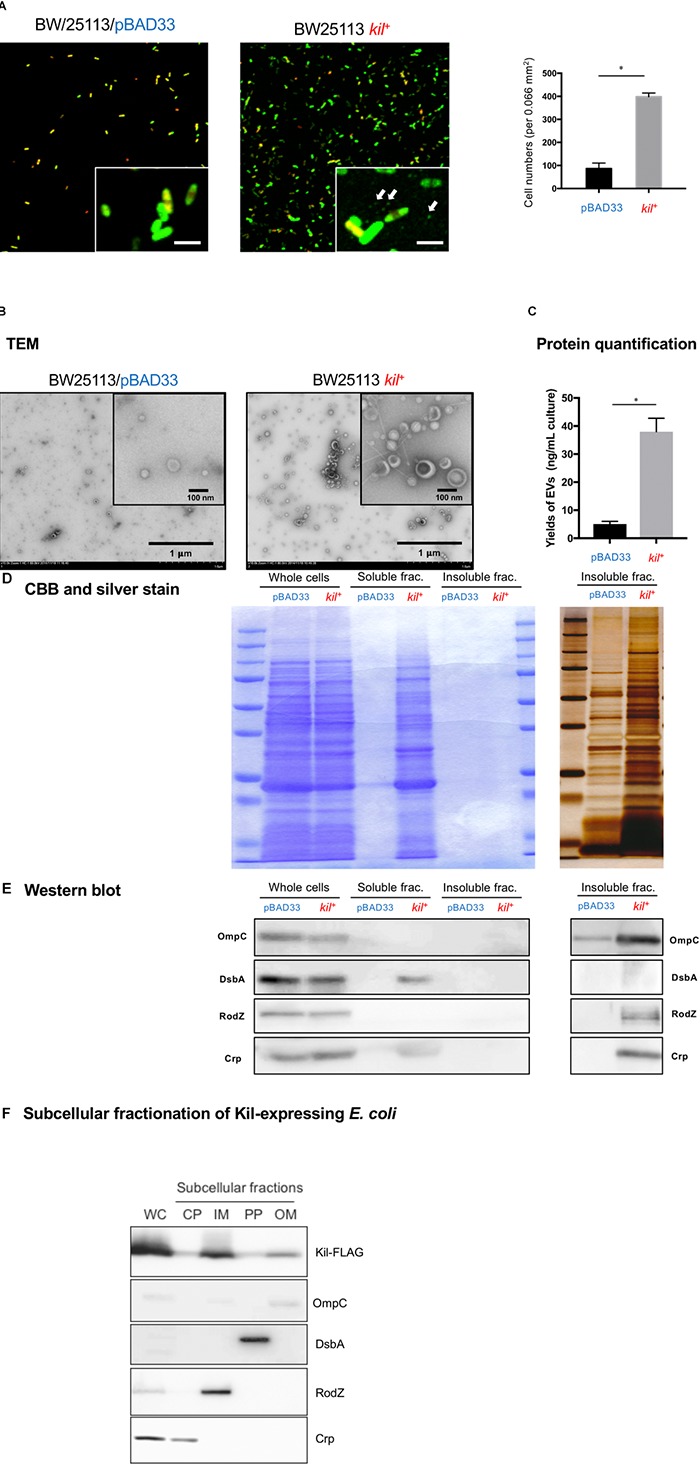
Characterization of MVs released from strains BW25113/pBAD33 and BW25113/pRN109 (*kil*^+^). **(A)** CFLM images of biofilms of BW25113/pBAD33 (left) and BW25113 *kil*^+^ (right). Both the strains were grown on coverslips in 24-well plates for 48 h under static conditions at 37°C in LB containing 0.02% arabinose and 5 mM MgSO_4_. The area of each image is 512 μm × 512 μm (*x* × *y*), and images with higher magnification are also shown in the inserts with 4-μm-long scales at the lower right of the electron microphotographs. The white arrows in the inset of BW25113 *kil*^+^ indicate presumed MVs. The numbers of bacterial cells adhered onto coverslips are also shown in the right bar graph. Attached cell numbers per a randomly selected area (0.066 mm^2^) on coverslips were counted. The results are expressed as the mean ± SD of three independent assays. **(B)** MVs isolated from 48-h broth cultures of BW25113/pBAD33 and BW25113 *kil*^+^ were subjected to TEM analysis following staining with uranyl acetate. Representative electron-micrographs are shown with 1-μm-long scale bars in the lower right corner. Images at higher magnification are shown in the inserts with 100-nm-long scale bars. **(C)** The protein amounts of MVs of BW25113/pBAD33 and BW25113 *kil*^+^ were quantified by Bradford assay. Data shown are the mean ± SD of three independent assays. Statistical analysis was performed using Mann-Whitney *U*-test. ^∗^*P* ≤ 0.05. **(D)** Protein profiles after SDS–PAGE of whole cells (whole cells), the supernatants (soluble frac.) (insoluble frac.), and the pellets after ultracentrifugation of 48-hour-old culture supernatants of BW25113/pBAD33 and BW25113 *kil*^+^ derivatives. Whole cell samples standardized at OD_600_ = 5, a 100-fold concentrated soluble fraction, and a 100-fold concentrated insoluble fraction were applied at 20 μl volume per lane of SDS–PAGE gels. Detection by CBB and silver staining were subsequently performed as indicated. **(E)** Western immunoblot detection of OmpC (outer membrane marker), DsbA (periplasmic marker), RodZ (inner membrane marker), and Crp (cytoplasmic marker) in same samples used in **(D)**. Results are shown in the left and right panels using different HRP substrate kits with high sensitivity (ECL Prime, GE Healthcare Bio-Sciences) and ultra-high sensitivity (Immobilon ECL Ultra, Millipore), respectively. **(F)** Subcellular localization of Kil in B25113/pRN132 cells. The whole cell lysate sample (WC) standardized at OD_600_ = 5 was applied at the volume of 20 μl per lane of SDS–PAGE gel. A 20-fold concentrated cytoplasmic fraction (CP), a 100-fold concentrated inner membrane fraction (IM), a 20-fold concentrated periplasmic fraction (PP), and a 100-fold concentrated outer membrane fraction (OM) were applied at 20 μl volume per lane in SDS–PAGE gels. Signals of outer membrane, periplasm, inner membrane, cytoplasm, and Kil were probed by antibodies against OmpC, DsbA, RodZ, Crp, and FLAG, respectively.

We also observed the biofilms by CLSM using SYTO-9/PI staining (Figure 5A). The attached cell numbers of BW25113 strain significantly increased by the introduction of the *kil* gene, whereas the ratio of membrane-damaged cell number to total attached cell number in case of the BW25113/*kil*^+^ strain was not different from that of the vector control stain (BW25113/pBAD33). Notably, small particles attached on the surface were observed in case of the BW25113 *kil*^+^ strain (the white arrows in the insets of Figure 5A) but not in the BW25113/pBAD33 strain. The data of CLSM showing the presence of small particles prompted us to look more in detail at the insoluble fraction of the bacterial culture supernatant by TEM (Figure 5B), Bradford analysis (Figure 5C), and protein profiling by SDS–PAGE (Figures 5D,E). TEM analysis revealed that BW25113 *kil*^+^ released large amounts of MVs with increase in the diameters, as compared with BW25113/pBAD33 (Figure 5B). We confirmed that MV release significantly increased in BW25113 *kil*^+^, as compared with BW25113/pBAD33 (Figure 5C). Protein profiling of bacterial supernatant by SDS–PAGE analysis and CBB staining and/or silver staining demonstrated that many protein bands could be detected in the soluble fraction of BW25113 *kil*^+^, as compared with BW25113/pBAD33 (Figure 5D). On a silver stained gel, more protein bands also appeared in insoluble fractions of BW25113 *kil*^+^, as compared with BW25113/pBAD33 (Figure 5D). Furthermore, immunoblot analysis using a series of antibodies detecting *E. coli* proteins revealed that all subcellular marker proteins (cytoplasmic, periplasmic, and outer and inner membranes) were included in insoluble fractions of BW25113 *kil*^+^, whereas only periplasmic and cytoplasmic proteins were detected in the soluble fraction (Figure 5E). On the other hand, in our subcellular fractionation study, Kil proteins were found to be localized at all subcellular fractions containing both inner and outer membranes (Figure 5F). Taken together, BW25113 *kil*^+^ released not only a soluble fraction but also insoluble inner and outer MVs into extracellular milieu.

### The *kil*^+^ BW25113 Showed Reduced Viability at a Stationary Phase and Increased Susceptibility to Killing by Predator Bacteria

**FIGURE 6:**
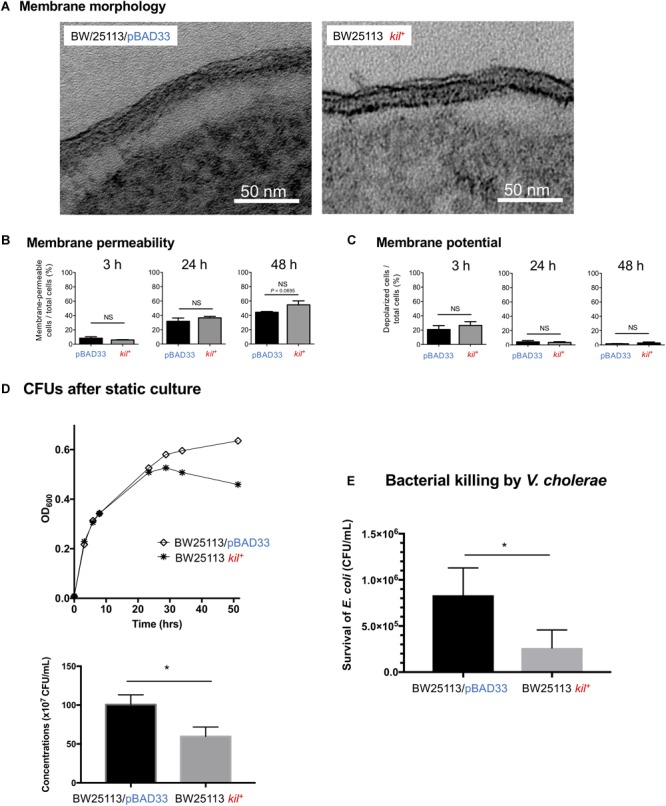
Growth and membrane integrity analysis of BW25113 *kil*^+^. **(A)** Thin-section TEM images of the envelope of BW25113/pBAD33 and BW25113 *kil^+^*. Bars, 50 nm. **(B)** Membrane permeability of BW25113/pBAD33 and BW25113 *kil^+^*. Membrane permeability of cells was defined as TO-PRO-3 iodide positive cells. Shown are “membrane-permeable cells/total cells (%)” at different time points (3, 24, and 48 h) after culture initiation. The results are expressed as the mean ± SD of three independent experiments, which appear in Supplementary Figure S5. Statistical analysis was performed using the Mann-Whitney *U*-test. NS, no significant difference. **(C)** Membrane potential of BW25113/pBAD33 and BW25113 *kil^+^*. Membrane depolarized cells and polarized cells were separated by fluorescence intensity of DiOC_2_(3). Shown are “depolarized cells/total TO-PRO-3 negative cells (%)” at different time points (3, 24, and 48 h) after culture initiation. The results are expressed as the mean ± SD of three independent experiments, which appear in Supplementary Figure S5. Statistical analysis was performed using the Mann-Whitney *U*-test. NS, no significant difference. **(D)** Strains BW25113/pBAD33 and BW25113 *kil^+^* were grown in LB media supplemented with 5 mM MgSO_4_, 0.02% arabinose under static conditions at 37°C. Absorbance at OD_600_ was measured at different time points during culture for 48 h (upper figure). Results of CFU counting after static culture for 48 h were expressed as the mean ± SD of three independent assays (lower figure). Statistical analysis was performed using the Mann-Whitney *U*-test. ^∗^*P* ≤ 0.05. **(E)** Susceptibility of strains BW25113/pBAD33 and BW25113 *kil^+^* to the T6SS-dependent killing effect by *V. cholerae* strain V52. Survival of the two *E. coli* strains (BW25113/pBAD33 and BW25113 *kil^+^*) was determined by measuring CFU/ml following exposure to *V. cholerae* strain V52. The results are expressed as the mean ± SD of four independent experiments. Statistical analysis was performed using the Mann-Whitney *U*-test. ^∗^*P* ≤ 0.05.

As far as we examined the membrane morphology, membrane permeability, and membrane potential, no significant difference was observed between BW25113/pBAD33 and BW25113 *kil*^+^ (Figures 6A–C and Supplementary Figure S5). Nevertheless, there was a tendency of increased membrane permeability at 48 h in BW25113 *kil*^+^ strain (*P* = 0.0695, when compared with the vector control (Figure 6B and Supplementary Figure S5). Then, we compared the growth behaviors of these strains (Figures 6D,E). In the growth curve analysis, the value of OD_600_ of BW25113 *kil*^+^ was almost equivalent to that of BW25113/pBAD33 until the late log phase; however, the value of the *kil*^+^ strain dropped after the stationary phase, but not the vector control (Figure 6D). The CFU counting at 48 h revealed that the viability of BW25113 *kil*^+^ was significantly lower than that of BW25113/pBAD33 (Figure 6D). We have also tested the susceptibility of these strains to killing by *V. cholerae* in a T6SS-dependent manner (Ishikawa et al., 2012). The results showed that BW25113 *kil*^+^ was more susceptible to the killing than BW25113/pBAD33 (Figure 6E). These data suggest that the *kil* gene inhibits the growth during the stationary phase and increases the susceptibility of BW25113 to interbacterial killing via T6SS without significant alteration in membrane morphology, membrane permeability, and membrane potential.

## Discussion

The plasmid pNTR-SD is a chimeric plasmid generated from pColE1 (Chan et al., 1985) and pJF118HF (Furste et al., 1986). The pNTR-SD has been commonly used as a parental plasmid of a complete set of mobile plasmid clones of intact open reading frames (ORFs) representing all the genes of *E. coli* K-12 (Saka et al., 2005). In the present study, we found that the *kil* gene in plasmid pNTR-SD was responsible for an increase in biofilm formation by *E. coli*. In the sequence analysis of pNTR-SD, the construct was found to lack the upstream sequence corresponding to the promoter region indispensable for the expression of *kil* gene (*cea-kil* operon) (Waleh and Johnson, 1985). Despite the absence of the natural promoter of the *cea-kil* operon, the *kil* gene in pNTR-SD was functionally active. Instead the *lacI*^q^ promoter was present at the upstream of the *kil* gene (see the plasmid map in Figure 3A), while no typical terminator was found in the intergenic region between the *lacI*^q^ and *kil* genes in the pNTR-SD plasmid. Therefore, we suggest that the *kil* gene on pNTR-SD is expressed by the transcriptional read-through from the promoter of *lacI*^q^.

**FIGURE 7:**
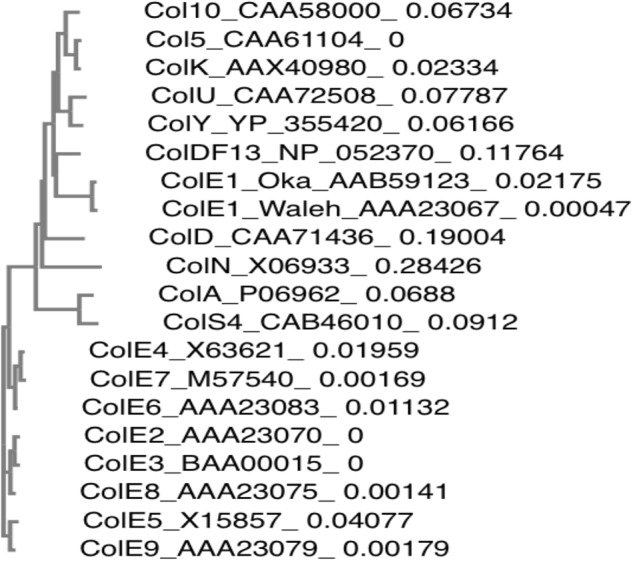
Phylogenetic distances between Kil protein homologs of naturally occurring colicinogenic plasmids. Phylogenetic tree constructed by the neighbor-joining method on the basis of the amino acid identity of Kil protein homologs among naturally occurring colicinogenic plasmids is shown. The protein accession numbers are denoted following the protein names. The branch lengths of the individual Kil homologs in the phylogenetic tree are the relative values of their phylogenetic distances, which are given following the respective protein accession numbers.

Besides pNTR-SD, several ColE1 derivatives unintentionally containing *kil* gene are commonly used as molecular genetics tools (Sugino and Morita, 1992; House et al., 2004; Scott et al., 2017). Therefore, we would like to call attention to a possible effect of the *kil* gene on enhancement of biofilm formation and release of proteinous materials including MVs, when the *kil*^+^ plasmid clones are used for a complementation or in overexpression studies. In addition, ColE1 and ColE1-like plasmids have been isolated from a wide range of species including some clinically important pathogens, such as extended-spectrum β-lactamase (ESBL)-producing *E. coli*, enteroaggregative Shiga toxin-producing *E. coli*, and *Shigella* spp. (Riley et al., 1994; Yang et al., 2005; Fricke et al., 2008; Holt et al., 2012; Kunne et al., 2012; Wang et al., 2014; Kurylo et al., 2016). In Table 3, natural and artificial ColE1 plasmid clones with information about the number of each identity and gap of the respective *kil* gene homologs when compared to the *kil* gene (138 nt) reported in 1979 are listed (Oka et al., 1979). In Figure 7, a phylogenetic tree was also constructed following the alignment of Kil protein homologs among naturally occurring colicinogenic plasmids. All the respective proteins derived from these plasmids were highly homologous to Kil from ColE1 (Oka et al., 1979). It has yet to be determined whether *kil* gene homologs in those natural occurring colicinogenic plasmids behave like the pNTR-SD or the *kil*-cloned plasmid (pRN109) used in this study. Nonetheless, the possibility should be addressed in future studies, because emergence and spread of bacteria that harbor plasmids with a *kil* locus in microbial communities might contribute to appearance of new pathoadaptive variants expressing the enhanced biofilm phenotypes. We therefore suggest that the possibility of horizontal transfer via these commonly occurring plasmids in diverse Enterobacteriaceae should be taken into account in the context of biofilm formation or protein/MV release into the extracellular milieu.

**Table 3:** Naturally occurring ColE1 clones and artificial plasmids containing *kil* gene of ColE1.

Plasmids	Relevant characteristics^a^	Sources	Identity^b^ % (nt nos.)	Gap^b^ % (nt nos.)
ColE1	ColE1 from *E. coli* isolates, 6.6 kb, ColE1 firstly reported in 1979.	Oka et al., 1979	100% (138/138)	0 (0/138)
ColE1	ColE1 from *E. coli* isolates, 6.6 kb, laboratory standard ColE1.	Waleh and Johnson, 1985	99% (137/138)	0 (0/138)
ColE1-EC12	ColE1 from human and animal *E. coli* isolates	Ochman and Selander, 1984; Riley et al., 1994	92% (127/138)	0 (0/138)
ColE1-EC24	ColE1 from human and animal *E. coli* isolates	Ochman and Selander, 1984; Riley et al., 1994	92% (127/138)	0 (0/138)
ColE1-EC31	ColE1 from human and animal *E. coli* isolates	Ochman and Selander, 1984; Riley et al., 1994	92% (127/138)	0 (0/138)
ColE1-EC39	ColE1 from human and animal *E. coli* isolates	Ochman and Selander, 1984; Riley et al., 1994	92% (127/138)	0 (0/138)
ColE1-EC40	ColE1 from human and animal *E. coli* isolates	Ochman and Selander, 1984; Riley et al., 1994	92% (127/138)	0 (0/138)
ColE1-EC50	ColE1 from human and animal *E. coli* isolates	Ochman and Selander, 1984; Riley et al., 1994	92% (127/138)	0 (0/138)
ColE1-EC71	ColE1 from human and animal *E. coli* isolates	Ochman and Selander, 1984; Riley et al., 1994	92% (127/138)	0 (0/138)
ColE1-MRE	ColE1 from a divergent *E. coli* strain MRE600 that displays phenotypes of the closely related *Shigella*; 7.1 kb	Kurylo et al., 2016	92% (127/138)	0 (0/138)
ColE1-H22	ColE1 from a probiotic *E. coli* strain H22; 7.1 kb	Kurylo et al., 2016	92% (127/138)	0 (0/138)
pH1519-7	Isolate from extended-spectrum β-lactamase (ESBL)-producing *E. coli*, 7.0 kb, Amp^r^	Wang et al., 2014	99% (137/138)	0 (0/138)
pSMS35_8	Isolate from multidrug-resistant environmental *E. coli* strain SMS-3-5, 8.9 kb	Fricke et al., 2008	92% (127/138)	0 (0/138)
pHUSEC41-4	Isolate from *Shigella sonnei* strain Ss046, 5.2 kb	Kunne et al., 2012	92% (127/138)	0 (0/138)
pSS046_spB	Isolate from a historical enteroaggregative Shiga toxin-producing *E. coli* strain HUSEC41, O104:H4, 5.2 kb	Yang et al., 2005	92% (127/138)	0 (0/138)
Plasmid B	Isolate from *S. sonnei* strain 53G, 5.2 kb	Holt et al., 2012	92% (127/138)	0 (0/138)
pNTR-SD	ColE1 derivative, 8.3 kb, Amp^r^	NIG collection (Japan)	99% (137/138)	0 (0/138)
pMK2016	ColE1 derivative, 7.0 kb, Spec^r^, Str^r^	House et al., 2004	100% (138/138)	0 (0/138)
pTS1	ColE1 derivative, 7.0 kb, Tet^r^	Scott et al., 2017	99% (137/138)	0 (0/138)
pMK20	ColE1 derivative, 4.1 kb, Km^r^	Prince and Jacoby, 1982	99% (137/138)	0 (0/138)
pMM234	ColE1 derivative, 9.1 kb, Neo^r^	Sugino and Morita, 1992	91% (126/138)	0 (0/138)

*^*a*^Amp^*r*^, ampicillin resistant; Neo^*r*^, neomycin resistant; Spec^*r*^, spectinomycin resistant; Str^*r*^, streptomycin resistant; Tet^*r*^, tetracycline resistant.*

*^*b,c*^Identities and gaps of the sequences of *kil* gene homologs when compared with the sequences of *kil* gene reported by Oka et al. (1979).*

In Figure 2A, we examined how pNTR-SD affects biofilm formation by a series of LPS mutant strains with different core oligosaccharide compositions. The results indicated that hyper-biofilm formation was occurring particular in the deep-rough LPS mutant strains, RN101 and RN102 (Figure 2). Thus, the mechanism by which the RN102/pNTR-SD strain enhanced biofilm formation might be involved in autolysis together with eDNA release due to its compromised outer membrane integrity. Alternatively, the enhanced biofilm formation by the deep-rough mutants harboring pNTR-SD may be due to the increased attachment of cell-to-cell or cell-to-abiotic surface via eDNA-mediated hydrophobic interaction. The view is because these deep rough strains showed very strong hydrophobicity at the cell surface (Nakao et al., 2012), and eDNA-mediated hydrophobic interaction is a key factor of the initial attachment mechanism in the biofilm studies of *Pseudomonas aeruginosa* and *Staphylococcus epidermidis* (Das et al., 2010, 2014).

The amino acid sequence of Kil is homologous to that of VirB7, one of the components of a type IV secretion system (T4SS) of *Agrobacterium tumefaciens* (Shirasu et al., 1990). In *A. tumefaciens*, the outer membrane lipoproteins VirB7 and VirB9 form outer membrane complex (OMC) together with a cell-envelope spanning unit VirB10. The OMC is intrinsically stable and stabilizing for most of the other subunits of T4SS. Morphology of the OMC has been visualized as a ring-like structure by TEM (Sarkar et al., 2013). These findings together with those from the functional analyses of the components of OMC suggest its contribution to substrate transfer by forming outer-membrane spanning pore. As shown in Supplementary Figure S6, a well-conserved “lipobox” motif was found in the sequences of both Kil and VirB7. However, neither sequence had Asp at position 2, which is known as the inner membrane retention signal (Cell 1988 Yamaguchi K). Thus, we predicted that not only VirB7, but also Kil, would translocate to outer membrane, but not to inner membrane. Nonetheless, in subcellular localization analysis, we found that the Kil proteins are localized at both the outer and inner membranes (Figure 5F). The reason for the unexpected result is under investigation. However, the subcellular localization of Kil at both the outer and inner membranes may attack both the membranes, resulting in the extracellular release of both outer and inner membrane proteins in *kil*-expressing strain.

Mg^2+^-enhanced biofilm formation of BW25113/pRN102 was found to be inhibited by protease in a dose-dependent manner (Figure 4D), indicating that both physiologically relevant concentrations of Mg^2+^ and proteinous materials released from the *kil*-expressing strain are indispensable for the enhanced biofilm formation. It has been also reported that Mg^2+^ promotes flagellation of *Vibrio fischeri* (O’Shea et al., 2005). Nevertheless, we could rule out the possibility that the enhanced biofilm formation was due to the overexpression of flagella or of Ag43 (Supplementary Figure S3). On the other hand, divalent cations have been shown previously to affect the viscoelastic properties of bacterial biofilms and stiffened the biofilms of *P. aeruginosa* (Jones et al., 2011). This may be true also in the case of *E. coli* in the presence of a physiologically relevant concentration of Mg^2+^. Alternatively, we propose that in Mg^2+^-supplemented media, a strong association of cell-to-cell or cell-to-the plastic surface may be mediated by the electrostatic interaction of Mg^2+^, as previously reported using motile and non-motile *P. aeruginosa* (Kerchove and Elimelech, 2008).

In clinical settings, the properties of bacterial biofilms in indwelling urinary catheters may be closely associated with the presence of Mg^2+^ in urine, because the most troublesome complications are crystalline biofilms composed of magnesium phosphate crystal as one of the principle components (Hedelin et al., 1984). The crystalline biofilms can occlude the catheter lumen and trigger episodes of pyelonephritis and septicemia (Stickler, 2008). Human urine contains Mg^2+^ at the concentrations ranging from 1 to 5 mM. It is suspected that the Mg^2+^ concentration is high near the crystalline biofilms and that bacteria can respond to the high concentration of Mg^2+^ there. We need to consider a possible role of a physiological relevant concentration of Mg^2+^ in the enhancement of biofilm formation in a medical setting, for example urinary catheter-associated infections.

Earlier reports indicate that Kil may alter the composition of envelope structures and cause release of outer membrane components such as LPS, phospholipids, and outer membrane proteins (Aono, 1989, 1991). It has also been reported that the *kil* gene enhanced the release of bacterial components into the extracellular milieu (Kobayashi et al., 1986; Aono, 1989; Miksch et al., 1997; Beshay et al., 2007). However, to the best of our knowledge, there is no previous description about membrane vesicle production induced by Kil. In the present study, we found that *kil* gene expression strongly induced MV production. There is accumulating evidence that MVs contribute to a variety of offensive or defensive functions of bacteria, *i.e.*, transport of toxins/antigens to host cells, attachment/biofilm formation, and immunomodulation through MV components such as ligands of Toll-like receptors (TLRs). So, if pathogenic or opportunistic bacteria release toxin-laden MVs due to a *kil*-expressing plasmid, the resultant excessive MVs may be a risk factor in clinical settings. As MVs were found to contribute to biofilm formation as well, enhanced release of MVs will presumably influence biofilm-associated infectious diseases. On the other hand, MVs are also regarded as the vehicle which can be applied as a therapeutic tool, *i.e.*, as cell-free immunogen/adjuvant for vaccination (Nakao et al., 2016; Schorey and Harding, 2016), vehicles of anticancer/anti-inflammatory drugs (Jain and Pillai, 2017; Kim et al., 2017). Accordingly, with respect to the bio-engineering applications, we suggest that the *kil*-expressing vector could be valuable for efficient isolation of larger amounts of MVs.

## Author Contributions

RN, SNW, and BEU significantly contributed to conception and design of the study. RN performed most of all experiments, analysis, and interpretation of data and wrote the first draft of the manuscript. SLM performed bacterial killing assay. All authors contributed to manuscript revision and read and approved the submitted version.

## Conflict of Interest Statement

The authors declare that the research was conducted in the absence of any commercial or financial relationships that could be construed as a potential conflict of interest.

## Footnotes

1 https://www.ebi.ac.uk/Tools/msa/tcoffee/
