# Supplementary material for: Enhanced Biofilm Formation and Membrane Vesicle Release by Escherichia coli Expressing a Commonly Occurring Plasmid Gene, kil
Source: Front Microbiol. 2018 Nov 7;9:2605. doi: 10.3389/fmicb.2018.02605 (PMC6234761; doi:10.3389/fmicb.2018.02605)
Supplement: Supplementary file 1 [file Data_Sheet_1.pdf]

Supplementary Materials

Enhanced biofilm formation and membrane vesicle release by *Escherichia coli* expressing a commonly occurring plasmid gene, *kil*

Ryoma Nakao[*, 1, 2], Si Lhyam Myint[1], Sun Nyunt Wai[1], and Bernt Eric Uhlin[*, 1]

[*]Correspondence:

Ryoma Nakao: ryoma73@nih.go.jp; Bernt Eric Uhlin: bernt.eric.uhlin@umu.se

1. Supplementary Text: Information of pRN132 Construction

Construction of pRN132

: pBAD33Ω [SD (14 nt)+*kil* gene (1(ATG)+45 aa) + Linker (10 aa)]

: *Eco*RI and *Kpn*I of pBAD33 were used for ligation of the fusion construct.

MCS sites (*Nhe*I, *Eco*RI, *Sac*I, and *Kpn*I) are still present at the upstream of SD (of *kil*) sequence.

SD sequence and the downstream: ATAAGGATCGAGTT

kil:
ATGAGGAAAAGATTTTTTGTGGGAATATTCGCGATAAACCTCCTTGTTGGA
TGTCAGGCTAACTATATACGTGATGTTCAGGGAGGGACCATCGCACCATC
CTCCTCTTCTAAACTGACGGGGATCGCGGTTCAG~~TAG~~

Linker: TCCTCCGCCGCCGTCAGCGGAATTACCGGT

*Bgl*II and *Sal*I site is present between Linker and FLAG tag sequence. [AGATCTGTC]

FLAG: GACTACAAGGACGATGACGACAAG

Resultant *kil*-FLAG fusion construct, which was confirmed by sequencing.

ATAAGGATCGAGTTATGAGGAAAAGATTTTTTGTGGGAATATTCGCGATAAAC
CTCCTTGTTGGATGTCAGGCTAACTATATACGTGATGTTCAGGGAGGGACC
ATCGCACCATCCTCCTCTTCTAAACTGACGGGGATCGCGGTTCAGTCCTCC
GCCGCCGTCAGCGGAATTACCGGTAGATCTGTCGACTACAAGGACGATGA
CGACAAGTGA

2. Supplementary Figures and Tables

2.1. Supplementary Figures

Supplementary Figure 1: Internalization of "cell-impermeant" dyes into the cells of RN102/pNTR-SD strains.

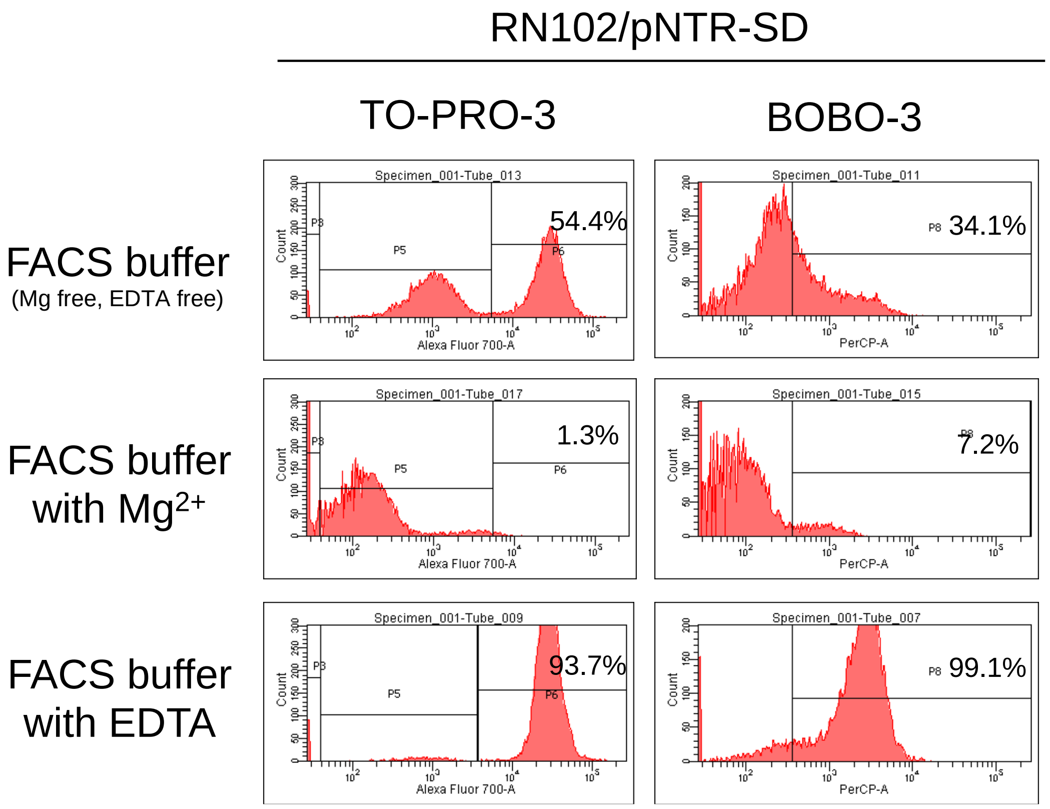

The presence of "leaky" cell population in the RN102/pNTR-SD cells. The RN102/pNTR-SD cells statically grown at 37 ºC for 3 hours was standardized at 1 x 10^6 CFU/mL in $MgSO_4$-free, EDTA-free FACS buffer (top panels), $MgSO_4$-supplemented FACS buffer (middle panels), or EDTA-supplemented FACS buffer (bottom panels). The $MgSO_4$-supplemented or EDTA-supplemented buffer was used to prepare a membrane integrity-recovered or -compromised control, respectively, because EDTA is known to increase permeability of bacterial cell membranes by chelating divalent cations such as Mg^{2+}, which substantially contribute to the membrane stabilization (Nikaido H. Microbiol Mol Biol Rev. 2003. 67(4): 593-656.). These samples were stained with 2 different "cell-impermeant" dyes; a TO-PRO-3 (excitation/emission of at 642/661 nm,

Thermo Fisher Scientific) at 1 μM or BOBO-3 (570/602 nm, Thermo Fisher Scientific) at 1 μM and subjected to flow cytometry analysis (FACS Canto II; BD Biosciences, Inc.). Of 10,000 events with each sample, real bacterial particles were discriminated from debris and noise using the forward scatter and side scatter channels (FSC/SSC), which was defined as total bacterial particles. Total bacterial particles were separated based on the difference in fluorescence intensity of TO-PRO-3 or BOBO-3 in a histogram. Shown are results of bacterial cells suspended with Mg^{2+}-free, EDTA-free buffer (top panels), 5 mM $MgSO_4$-supplemented buffer (bottom panels) 1 mM EDTA-supplemented buffer (bottom panels) with the percentages of TO-PRO-3-positive (left panels) or BOBO-3-positive (right panels) cell population. The data shown are representative of 3 independent experiments and similar results were obtained in other 2 experiments.

Supplementary Figure 2: Effect of *kil* gene expression on biofilm formation of BW25113 and Δ*pldA* strains.

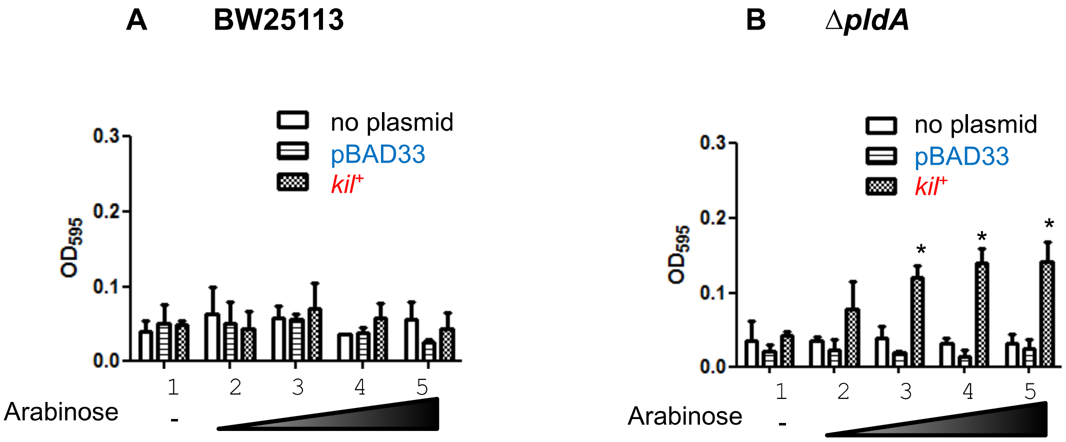

Bacterial strains were statically grown for 48 hours in a 96-well polystyrene plate. Biofilms were visualized by staining with 0.1% crystal violet. (A) Biofilm formation of BW25113, BW25113/pBAD33, and BW25113 *kil*$^{+}$ in the absence or presence of arabinose at different concentrations. (B) Biofilm formation of Δ*pldA*, Δ*pldA*/pBAD33, and Δ*pldA kil*$^{+}$ in the absence or presence of arabinose at different concentrations. (A and B) Biofilms were visualized by staining with 0.1% crystal violet. Lane 1, no addition of arabinose; Lane 2, 0.0002% arabinose; Lane 3, 0.002% arabinose; Lane 4, 0.02% arabinose; Lane 5, 0.2% arabinose. The mean ± SD of results from 3 independent experiments are shown. Statistical analysis was performed by Mann-Whitney U test. $*P \leq 0.05$, when comparing biofilm formation levels between the strains harboring an empty vector (pBAD33) and the *kil*-expression vector (*kil*$^{+}$) at the same concentration of arabinose.

Supplementary Figure 3: The effect of Mg^{2+} on expression of FliC and Ag43 by strain BW25113/pRN109.

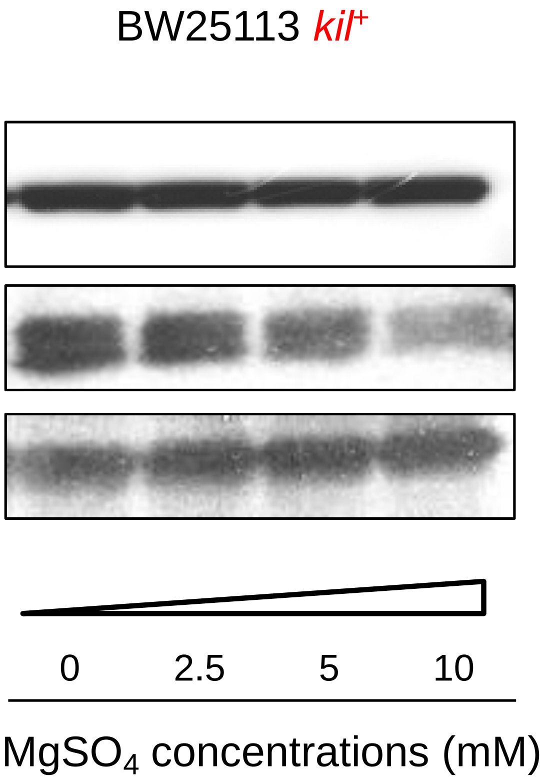

Expression levels of the flagella protein FliC, and of the surface protein Ag43 in BW25113/pRN109 grown for 48 hours at 37°C in LB containing 0.02% arabinose and different concentrations of $MgSO_4$ ranging from 0 to 10 mM. Detection of the major outer membrane protein OmpC was used as a loading control for the sample set. The whole cells samples were subjected to Western immunoblot analysis using antisera against FliC, Ag43, and OmpC. For detection of immunoreaction with the 2nd antibody labeled with HRP, the ECL prime kit (GE Healthcare Bio-Sciences) was used.

Supplementary Figure 4: The effect of DNase I and Protease on biofilm formation of a *kil*-expressing flagella mutant strain.

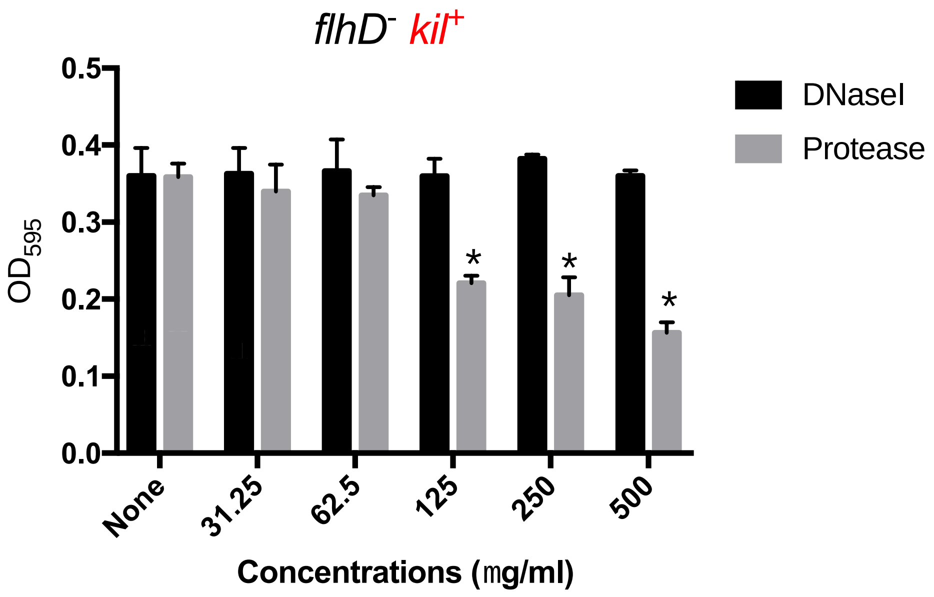

Biofilm formation by a flagella mutant harboring *kil* plasmid (*flhD*$^-$ *kil*$^+$) in the presence of 5 mM $MgSO_4$ with DNase I or protease at different concentrations. DNase I and protease were used at the following concentrations: lane 1, 31.25 μg/ml; lane 2, 62.5 μg/ml; lane 3, 125 μg/ml; lane 4, 250 μg/ml; lane 5, 500 μg/ml. The results are expressed as the mean ± SD of 3 independent assays. Statistical analysis was performed using Mann-Whitney U-test. *$P \leq 0.05$, when compared with the case without treatment (None).

Supplementary Figure 5: Evaluation of Membrane permeability and membrane potential of whole cells of BW25113 strains

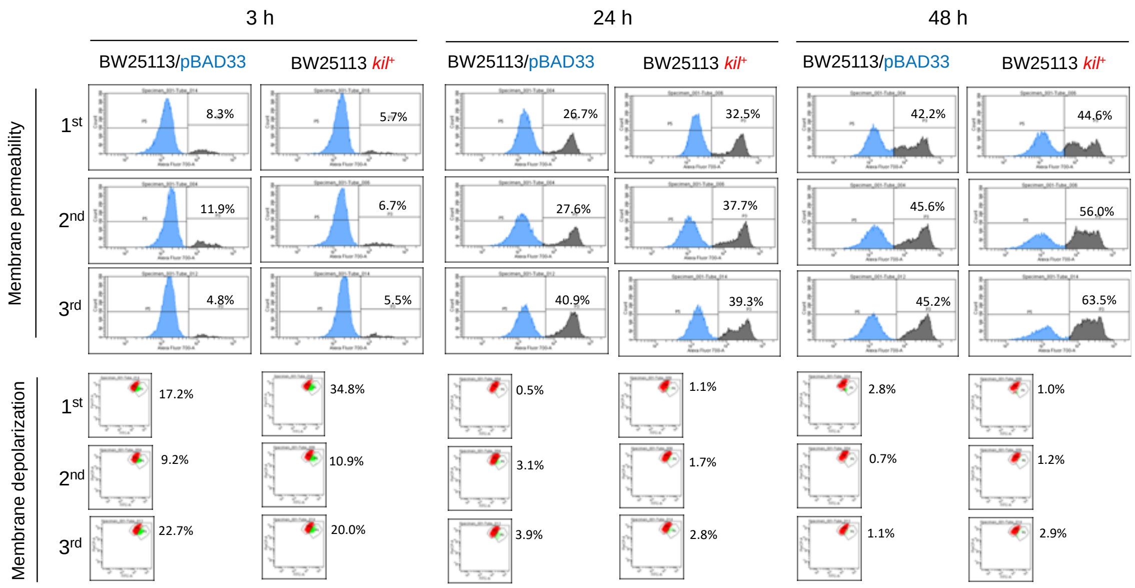

Three independent experiments data from FACS analysis of Fig. 6(B) and (C). Total bacterial particles were separated based on the difference in TO-PRO-3 fluorescence intensity in a histogram (Membrane permeability: top panels) and the membrane integrity-compromised cell population was defined by referring to a heated sample shown to be TO-PRO-3-positive (data not shown). Percentages of membrane-compromised cells (black-colored population in top panels) were indicated in each histogram. The TO-PRO-3-negative cell population was further divided into 2 subpopulations; polarized and depolarized cell populations in a two-dimensional dot plot (Membrane depolarization: bottom panels) by referring to a sample treated with CCCP as an ionophore control to induce membrane depolarization without increased membrane permeability (data not shown). Percentages of membrane-depolarized cells (green-colored population in bottom panels) were indicated at the upper right of each dot plot.

Supplementary Figure 6: Alignment of the predicted amino acid sequences of VirB7 and Kil precursors.

```
                               123•••
Af VirB7      ---MKYCLLCLVVALSGCQTNDTLASCKGPIFPLNVGRWQPTPSDLQLGNSGGRYDGA
Ec_ColE1_kil  MRKRFFVGIFAINLLVGCQANYIRDVQGGTIAPSSSS--KLTGIAVQ-----------
                   :   :  : * ***:*       *.* * . .  : *   :*
```

ClustalW alignment between the predicted amino acid sequences of VirB7 of *Agrobacterium tumefaciens* TiC58 plasmid (55 aa, Protein accession no. PJW24147) and Kil of *E. coli* ColE1 plasmid (45 aa, AAZ50524). The underlining or yellow highlighting represent sequences of potential signal peptide and lipobox, respectively.

'*' indicates positions which have a single, fully conserved residue and

':' indicates that one of the following 'strong' groups is fully conserved:

STA
NEQK
NHQK
NDEQ
QHRK
MILV
MILF
HY
FYW

'.' indicates that one of the following 'weaker' groups is fully conserved:

CSA
ATV
SAG
STNK
STPA
SGND
SNDEQK
NDEQHK
NEQHRK
FVLIM
HFY

Supplementary Figure 7: Absorbance spectra of crystal violet solution in ethanol.

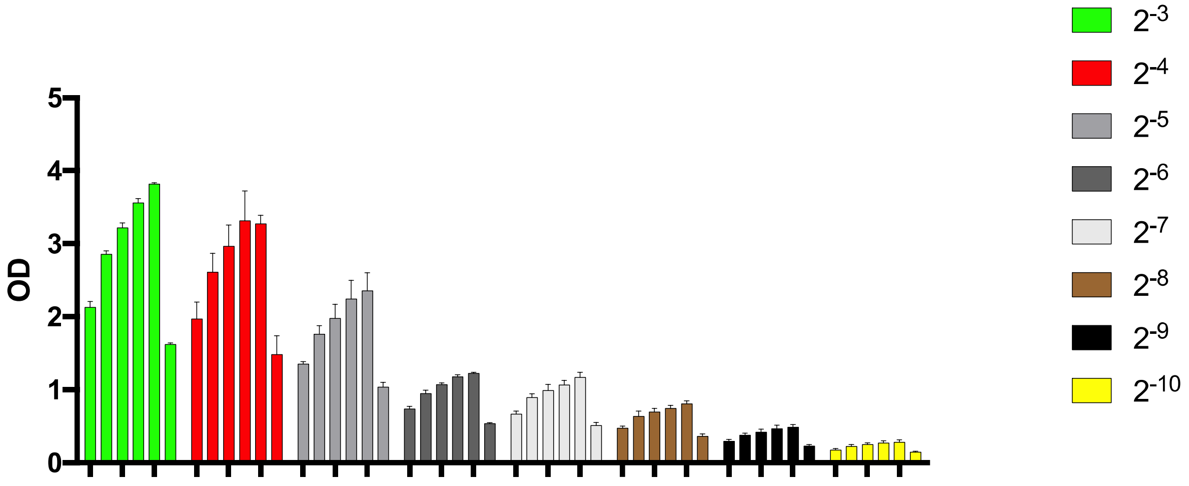

The solution of 0.1% crystal violet was 2-fold serially diluted (2^{-3} to 2^{-10}), and measured at 6 different ODs in the following order from left: 530, 545, 560, 575, 595, and 615 nm. Data is shown as mean ± SD of a triplicate assay. This experiment was repeated once again and similar results were obtained.

To examine the maximum absorbance of the CV solution, absorbance was measured at different ODs. The 0.1% CV solution was two-fold serially diluted with ethanol. Maximum absorbance appeared at 575 or 595 nm. Absorbance at 595 nm was chosen for the CV stain-based biofilm formation assay.

2.2. Supplementary Table

Supplementary Table 1 Oligonucleotides used in this study

Oligonuculeotide	Sequence (5'-3')[a]	Usage
HindIII-Mob-F	CCCAAGCTTatctgtgcggcatttcacac	cloning of *mob* operon
BamHI-Mob-R	CGGGATCCgcggtttccattataa	cloning of *mob* operon
kil2-f	ataaggatcgagttatgagg	cloning of *kil* gene
kil2-r	ctactgaaccgcgatccccg	cloning of *kil* gene
M13-f	gtaaaacgacggccag	primer pairs flanking multiple cloning sites of pMD20-T
M13-r	caggaaacagctatgac	primer pairs flanking multiple cloning sites of pMD20-T
pBAD-f1	ctttgctatgccatagcatt	primer pairs flanking multiple cloning sites of pBAD33
pBAD-r1	ctactgccgccaggcaaatt	primer pairs flanking multiple cloning sites of pBAD33
traA-f	agtgttcagggtgcttctgc	detection of *traA* gene
traA-2	tcagaggccaacgacggcca	detection of *traA* gene

[a] Restriction enzyme sites incorporated into oligonucleotides for subcloning are capitalized.
